# Enzymatic Responses to Low-Intensity Radiation of Tritium

**DOI:** 10.3390/ijms21228464

**Published:** 2020-11-11

**Authors:** Tatiana V. Rozhko, Elena V. Nemtseva, Maria V. Gardt, Alexander V. Raikov, Albert E. Lisitsa, Gennadii A. Badun, Nadezhda S. Kudryasheva

**Affiliations:** 1 Department of Medical and Biological Physics, Krasnoyarsk State Medical Academy, 660022 Krasnoyarsk, Russia; 2 Biophysics Department, Siberian Federal University, 660041 Krasnoyarsk, Russia; enemtseva@sfu-kras.ru (E.V.N.); gardt1998@list.ru (M.V.G.); inertnost@gmail.com (A.V.R.); alisitsa@sfu-kras.ru (A.E.L.); n-qdr@yandex.ru (N.S.K.); 3 Institute of Biophysics SB RAS, FRC KSC SB RAS, 660036 Krasnoyarsk, Russia; 4 Department of Chemistry, Moscow State University, 119991 Moscow, Russia; badunga@yandex.ru

**Keywords:** hormesis, low-dose radiation, tritium, enzymes, bacterial luciferase, oxidoreductase, fluorescent protein

## Abstract

The present study considers a possible role of enzymatic reactions in the adaptive response of cells to the beta-emitting radionuclide tritium under conditions of low-dose exposures. Effects of tritiated water (HTO) on the reactions of bacterial luciferase and NAD(P)H:FMN-oxidoreductase, as well as a coupled system of these two reactions, were studied at radioactivity concentrations ≤ 200 MBq/L. Additionally, one of the simplest enzymatic reactions, photobiochemical proton transfer in Coelenteramide-containing Fluorescent Protein (CLM-FP), was also investigated. We found that HTO increased the activity of NAD(P)H:FMN-oxidoreductase at the initial stage of its reaction (by up to 230%); however, a rise of luciferase activity was moderate (<20%). The CLM-FP samples did not show any increase in the rate of the photobiochemical proton transfer under the exposure to HTO. The responses of the enzyme systems were compared to the ‘hormetic’ response of luminous marine bacterial cells studied earlier. We conclude that (1) the oxidoreductase reaction contributes significantly to the activation of the coupled enzyme system and bacterial cells by tritium, and (2) an increase in the organization level of biological systems promotes the hormesis phenomenon.

## 1. Introduction

All organisms on Earth are adapted to the natural background radiation. Natural or/and anthropogenic incidents that resulted in an increased background radiation might induce different radiobiological effects: the radiotoxic suppression of physiological functions or an adaptive response. The latter usually occurs in the form of the activation of organismal physiological functions and takes place under a low-dose irradiation.

Health risks for exposures to a low-dose ionizing radiation remain controversial and are still a subject of intensive debates. The relevance of risk assessment criteria should be based on the molecular mechanisms of the biological responses; this approach is recognized worldwide [1], providing important tasks for radiation medicine and modern pharmacology. Modern medicine intensively develops therapeutic methods based on low-dose radiation; these include therapy for rheumatoid arthritis [2], prevention of tumor lesion growth [3], and a concomitant therapy for bacterial infections [4]. Understanding the molecular mechanisms can provide a tool for regulating the cellular response to radiation exposure [5].

Activation by a low-dose impact is a common phenomenon; it is associated with the term “hormesis”, which usually implies a favorable biological response to a low dose of toxins, radiation, or other stress factors. The hormesis phenomenon was described for the first time by H. Schulz and R. Arndt at the end of the 19th century [6]. The current resurgence of the hormesis concept refers back to Edward Calabrese [7,8,9,10,11]. The term ‘radiation hormesis’ was suggested by T.D. Luckey [12]. The phenomenon of radiation hormesis has been intensively studied [8,13,14].

Hormesis is considered as a basis for the most general toxicological model, which involves opposite response types: the activation of physiological functions under low-dose exposures and their inhibition by higher doses. Two other toxicological models, linear and threshold, might be considered as particular cases of hormesis [8,15,16,17]. It is supposed that hormesis is a highly general phenomenon that is independent from the biological model and the level of organization of a biological system (e.g., cell, organ, or organism) [9]. Studies of cellular responses form a basis for understanding low-dose effects on multicellular organisms. The role of enzymatic processes in the responses of living organisms to low-dose exposures is yet not fully understood.

Luminous marine bacteria and their enzyme reactions are highly convenient objects for studying and comparing the bioeffects of low-dose radiation at the cellular and enzymatic levels. The reasons for this are as follows.

(1) Marine bacteria [18,19,20,21] and their enzymes [22] have been used as bioassays for several decades; this is why the effects of exogenous compounds on these assay systems have been intensively studied. The effects of a series of radionuclides [23,24,25,26] and gamma radiation [27,28] on the bacteria and their enzymatic reactions were studied and compared. Thus, the predictive premise for the bioassays was formed based on the molecular mechanisms of the radiation-induced effects.
(2) Bioluminescence intensity is a tested physiological parameter under monitoring. The registration of luminescence is a convenient bioassay procedure; its advantages are a high sensitivity, high rates (duration down to 1–3 min), simplicity, as well as the availability of reagents and instruments. The high rates adapt the tests for a large number of measurements under comparable conditions and hence for a proper statistical processing, which is extremely important for low-dose exposures usually described in terms of “stochastic effects” [29]. Furthermore, the quick luminescence response assumes a nongenetic mechanism of low-intensity effects [30,31].

We used luminous marine bacteria to monitor low-dose radiation effects for the first time in [23], with the alpha-emitting radionuclide americium-241 as an example. Later, the effects of the alpha- and beta-emitting radionuclides americium-241, uranium-235 + 238, and tritium were additionally investigated [24,26,32]. We demonstrated that the bioluminescence response of the bacteria to the radionuclides included three stages: (1) threshold, (2) activation, and (3) inhibition. Such a complex response was described in terms of the hormesis model. A signaling role of reactive oxygen species in the low-dose effects of tritium and americium-241 was considered in [31,32,33].

In the current study, we have chosen tritium as a model beta-emitting radionuclide to compare low-dose radiation effects on cells and enzymatic reactions. The choice was justified by the environmental occurrence of tritium as the result of its natural and anthropogenic origin: on the one hand, this isotope is generated in the top layers of the atmosphere because of the space irradiation; on the other hand, tritium is a byproduct of many processes in the nuclear industry, and its local increase occurs around nuclear power plants and rises dramatically after nuclear incidents.

The total energy of tritium beta-decay is low (18.6 keV), and the average energy of electrons is low as well (5.7 keV). This is the reason for considering tritium as one of the less hazardous radioisotopes. The products of the decay of tritium (T) include an electron (a beta particle) and an ionized isotope of helium (${}_{2}^{3}{He}^{+}$):

(1) $$T\overset{\beta }{\to }{}_{2}^{3}{He}^{+}+e^{-}+\tilde{\nu }$$

**Figure 1:**
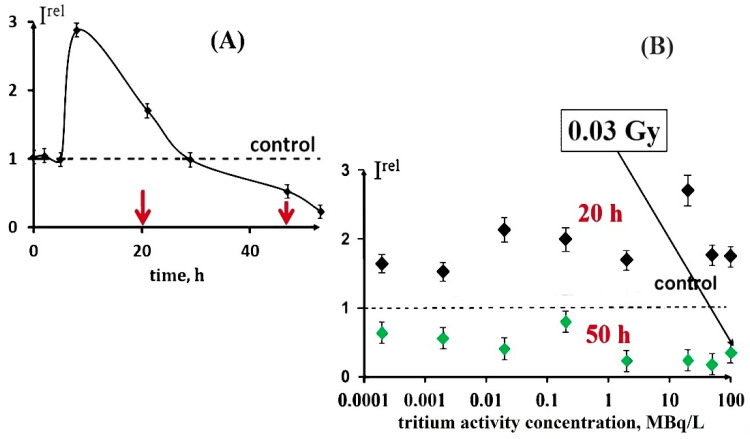
Effect of tritiated water on bioluminescence of bacteria. (**A**) Bioluminescence kinetics of bacteria in tritiated water, 2 MBq/L; red arrows denote times of sampling (20 and 50 h); (**B**) bioluminescence intensity vs. activity concentration of tritiated water at 20 h (black) and 50 h (green) exposures [16]. Adapted from Kudryasheva, N.S.; Kovel, E.S. Monitoring of low-intensity exposures via luminescent bioassays of different complexity: Cells, enzyme reactions, and fluorescent proteins. *Int. J. Mol. Sci.* **2019**, *20*, 4451.

These decay products are able to trigger electron/charge transfer in biochemical reactions and hence to affect the rates of cellular processes. Our previous studies [24,25] demonstrated both activation and inhibition effects of tritium on marine bacteria (Figure 1A), as well as the absence of a monotonic dependence of a luminescence response vs. the tritium activity concentration at a chronic low-dose exposure (<0.03 Gy) in a wide range of tritium radioactivities: from 0.0001 to 200 MBq/L (Figure 1B). This result was explained with the hormesis model involved in terms of the adaptation ability of the bacterial cells to the low-dose radiation.

An increase in the bacterial luminescence intensity in the presence of tritiated water, HTO, was demonstrated in a series of experiments. The biphasic dependence (activation + inhibition) was found in [24,26], while the monophasic dependence (activation only) was shown in [30,32,33].

The mechanism of the activation effect of tritium is a question of special interest. The first hypothetical mechanism is based on repairing DNA damage [28,34,35]. The involvement of nongenetic mechanisms in low-dose chronic radioactive effects in bacteria was proved earlier in [30,33]. Recently [32], we observed a 300% activation of bacterial bioluminescence by HTO; it was attributed to a “bystander effect” and explained by the ‘trigger’ function of the tritium radioactive decay products, as well as by a signaling role of reactive oxygen species.

An important way to explore the mechanisms of low-dose responses is based on the comparison of responses from biological structures of different complexities—a multicellular organism, cells, enzymatic reactions, etc. The effects of low-dose radiation on enzymatic processes and their comparison with those on cells is currently a very important focus. Previously, low-intensity exposures were investigated using the enzymatic bioassay—a system of coupled bacterial enzymatic reactions [23,24]. However, the enzymatic activity in individual biochemical reactions has not been studied yet.

The current study elucidates the low-dose effects of tritium in individual (i.e., noncoupled) enzyme reactions. Enzymatic reactions of bacterial luciferase and NAD(P)H:FMN-oxidoreductase were chosen. We compared the tritium effects on the individual enzymatic reactions with those on the coupled system of these reactions and on bacterial cells.

Additionally, one of the simplest enzymatic bioassay systems, i.e., a Coelenteramide-containing Fluorescent Protein (CLM-FP), was used to evaluate the activation bioeffect of tritium. CLM-FPs are products of bioluminescent reactions of coelenterates that constitute a potential use in biomedicine as biomarkers [36,37,38]. The fluorescence characteristics of CLM-FPs were intensively studied [39,40,41,42,43,44,45,46]; they were found to be sensitive to external exposures: excitation energy [44], chemical agents [47,48], and radioactivity [49,50]. Recently, [51] suggested CLM-FPs for intracellular application as being the simplest toxicity bioassay.

**Figure 2:**
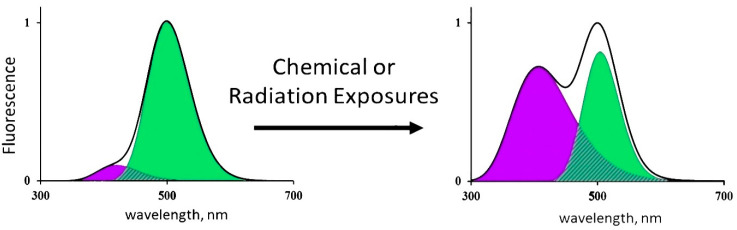
Changes in the fluorescence spectrum of CLM-FP exposed to chemical agents or radiation [16]. Adapted from Kudryasheva, N.S.; Kovel, E.S. Monitoring of low-intensity exposures via luminescent bioassays of different complexity: Cells, enzyme reactions, and fluorescent proteins. *Int. J. Mol. Sci.* **2019**, *20*, 4451.

The CLM-FP-based bioassay applies the simplest enzymatic reaction, i.e., photochemical proton transfer; its efficiency depends on the structure of the CLM-FP complex. Any destructive exposures, chemical or radioactive, can change this efficiency and, therefore, change contributions of the components of the reaction—protonated and deprotonated forms of CLM. Hence, as a result of the CLM-FP photoexitation, one can observe changes in the fluorescence contributions of the protonated (violet) and deprotonated (green) forms of CLM. An illustration of such changes is presented in Figure 2.

Previously, a low-dose exposure of the CLM-FP to tritium radiation did not reveal the intensification of the photobiochemical proton transfer; only inhibition was observed [49]. However, a variation of the initial characteristics of the CLM-FP preparation might change its sensitivity to the radioactive impact.

Our studies of enzymatic responses can provide a more detailed understanding of the mechanisms of low-dose effects and expand the application of enzyme systems for the diagnosis and/or regulation of intracellular processes. For example, bioluminescent reactions of coelenterates are widely used as genetically encoded markers in biological and medical research [36,37,38], and the parallel use of the products of their reactions (CLM-FPs) to assess the radiation-induced processes in the intracellular media would provide these reagents with multifunctionality.

A prospect for evaluating the radiotoxic or radioprotective response can occur for luminous bacteria, systems of their coupled enzymatic reactions [16,25], as well as for individual enzymatic reactions.

The current report compares the activation effects of tritium in luminous marine bacteria and coupled enzymatic reactions (Section 2.1) with those in individual enzymatic reactions catalyzed by bacterial luciferase (Section 2.2), NAD(P)H:FMN-oxidoreductase (Section 2.3), and different preparations of CLM-FP (Section 2.4). By this, we compare the sensitivity of the biological systems of different levels of organization (cells, a coupled enzyme system, individual enzyme reactions of two types, and the photobiochemical process of proton transfer) to the chronic low-dose tritium irradiation. The study develops the approach [16] for comparing the responses of biological systems of different complexities to low-intensity factors.

## 2. Experimental Section

Tritiated water (HTO) of radiochemical purity 98% was used as a source of beta-type ionizing radiation. The effects of tritium on four enzymatic systems were studied: the reaction of bacterial luciferase (Section 2.1), NAD(P)H:FMN-oxidoreductase (Section 2.2), a coupled system of two enzymatic reactions: bacterial luciferase–NAD(P)H:FMN-oxidoreductase (Section 2.3), and the photoluminescence of Coelenteramide-containing Fluorescent Protein (CLM-FP) (Section 2.4).

### 2.1. Reaction of Bacterial Luciferase. Reagents, Procedure, and Data Analysis

The reaction catalyzed by bacterial luciferase is shown below:

(R1) $$\mathrm{FMN}\cdot H^{-}+\mathrm{RCHO}+O_{2}\overset{\mathrm{luciferase}}{\to }\mathrm{FMN}+{\mathrm{RCOO}}^{-}+H_{2}O+hv$$

Reagents: bacterial luciferase from *Photobacterium leiognathi* (99% purity) was purchased from Biolumdiagnostika Ltd. (Russia). FMN (Sigma) and decanal (Acros Organics) were of analytical grade; EDTA (ROTH) and NaCl (Khimreactiv, Russia) were of chemical grade. The freshly prepared stock solution of 2 × 10^−2^ M decanal in ethanol was used. All the other reagents were dissolved in 0.05 M phosphate buffer, pH 6.8. A set of HTO samples of 0.0001−200 MBq/L specific radioactivity was used for adding to bacterial luciferase; the samples were prepared by mixing different volumes of the stock HTO solution with 3% sodium chloride.

The activity of bacterial luciferase was determined in a single-turnover reaction using the stopped-flow technique [52]. A buffer solution containing 3 × 10^−5^ M FMN and 10^−2^ M EDTA (solution A) was made anaerobic by bubbling with argon for 5 min. Photoreduction of FMN was carried out by exposure to the light of an incandescent lamp for 5 min. A separate air-equilibrated buffer solution of 2 × 10^−6^ M bacterial luciferase was incubated for 5 min with HTO of various specific radioactivities or 3% sodium chloride (control experiment). Then, a small volume of decanal solution was added to get a 6 × 10^−5^ M concentration (solution B). The stopped-flow experiment was initiated by rapid mixing of 75 μL solution A and 75 μL solution B at 20 °C using an SX-20 analyzer (Applied Photophysics). The kinetics of the bioluminescence intensity was recorded for 15 s with a photomultiplier directly from the observation cell without additional filters. All experiments were carried out in three replications.

To evaluate the luciferase activity, the value of the quantum yield, *Q*, was calculated. The *Q*-values evaluate the number of quanta emitted per single turnover of the enzyme; they were estimated as an area under the kinetic curve for all specific HTO radioactivities. The relative quantum yields, *Q^rel^*, were calculated as a ratio of the *Q*-values after incubation in the radioactive solutions to those in the control (nonradioactive) solutions. The average *Q^rel^* values and experimental errors were evaluated.

Additionally, the luciferase preparation was exposed to HTO (2 MBq/L) for 0.08, 0.43, 0.76, 1.5, 2.5, 20, 29, and 87.5 h; the enzyme activity was determined at the different times of exposure.

### 2.2. Effect of Tritium on the Reaction of NAD(P)H:FMN-Oxidoreductase. Reagents, Procedure, and Data Analysis

The reaction catalyzed by NAD(P)H:FMN-oxidoreductase is shown below [19]:

(R2) $$\mathrm{NADH}+\mathrm{FMN}\overset{\mathrm{NAD}\left(P\right)H:\text{FMN-oxidoreductase}}{\to }\mathrm{FMN}\cdot H^{-}+{\mathrm{NAD}}^{+}$$

NAD(P)H:FMN-oxidoreductase was obtained from the Photobiology lab, Institute of Biophysics, SB RAS, Krasnoyarsk, Russia; FMN was from SERVA, Germany; NADH was from ICN, USA. To construct the reaction mixture, we used the oxidoreductase solution (0.15 activity units), 10^−4^ M FMN, 10^−4^ M NADH. The specific radioactivity of HTO varied in the range of 0.0002 to 200 MBq/L. The reaction was performed in 0.05 M phosphate buffer, pH 6.8, at 20 °C.

A double beam UV/VIS spectrophotometer UNIKON-943, Italy, was used to measure the optical density and enzymatic activity of NAD(P)H:FMN-oxidoreductase. The registration wavelength (340 nm) corresponded to the NADH absorption maximum. The enzymatic activity was estimated as an angular coefficient in the exponential dependence of the optical density on time; it was determined using the built-in program. The reliability of the determination was controlled by the coefficient of approximation R^2^ being within the range of 0.98–0.99.

The enzymatic activity of NAD(P)H:FMN-oxidoreductase was studied in the control and radioactive solutions in five parallel experiments for different HTO specific radioactivities; the relative enzymatic activity was calculated and plotted vs. (1) the time of exposure and (2) the specific radioactivity of the solutions at 0 and 110 min of exposure. Experimental errors for the relative experimental activity did not exceed 10%.

### 2.3. Coupled System of Two Enzymatic Reactions: Bacterial Luciferase–NAD(P)H:FMN-Oxidoreductase. Procedure and Data Analysis

The reactions of bacterial luciferase and NAD(P)H:FMN-oxidoreductase are presented above (R1 and R2, respectively); enzymes and reagents are described in Section 2.1 and Section 2.2. Reaction mixtures are presented in [24]. The standard procedure for bioluminescence measurements was described earlier [24].

The bioluminescence intensity was registered with a microplate luminometer Luminoskan Ascent (Thermal Fisher Corp.) at 20 °C. Bioluminescence kinetics was recorded in control (nonradioactive) and radioactive samples of different specific radioactivities (≤200 MBq/L). The time of the exposure was restricted to 2 h because of the bioluminescence decay in the control samples. The experiments were conducted in four replications. The bioluminescence intensity in samples with different radioactivities was compared to that in the control samples; the relative bioluminescence intensities, *I^rel^*, were calculated. Experimental errors for *I^rel^* did not exceed 10%.

### 2.4. Effect of Tritium on Photoluminescence of CLM-FP Reagents, Procedure, and Data Analysis

To construct a bioassay system based on CLM-FP, the recombinant preparation of the photoprotein obelin from the hydroid polyp *Obelia longissima* was applied. The preparation was obtained from the Photobiology Lab, Institute of Biophysics, SB RAS, Krasnoyarsk, Russia [53]. Tris and ethanol were obtained from Fluka, Switzerland; EDTA from Sigma, Germany.

The detailed methodology of the experiment was described in [49]. HTO was added to the CLM-FP solutions. The characteristics of the sample solutions were: 200 MBq/L specific radioactivity and 10^−5^ M obelin concentration. The overall time of exposure to HTO was 19 days; t = 20 °C.

The fluorescent spectra were registered with a Cary Eclipse Fluorescence Spectrophotometer, Agilent, USA, at 350 nm photoexcitation, 20 °C. The fluorescence quantum yields *Q* were calculated in the coordinates: fluorescence intensity vs. wavelength number.

The complex fluorescence spectra of CLM-FP were deconvolved into individual components using the Gaussian distribution with mathematical processing by the software packages *OriginPro 2018 SR1 b9.5.1.195* and *Excel 2010*. To determine the maxima and number of the spectral components, we used the method of the second derivative. The deviation *d* of the calculated spectrum from the experimental one was evaluated as:

(2) $$d=\frac{\left|S_{\exp}-\sum S_{comp}\right|}{S_{\exp}}\cdot 100\%,$$

where *S*_exp_ is the area of the overall experimental spectrum, and *S_comp_* is the area of the individual spectral components. The value of *d* did not exceed 0.5%.

The contributions *W* of the ‘violet’ or ‘blue-green’ spectral components to the overall fluorescence spectrum were calculated as follows:

(3) $$W=\frac{S_{comp}}{\sum S_{comp}}.$$

The values of *Q* and *W* were obtained in two parallel 19-day experiments with three measurements for all irradiated and control (nonradioactive) CLM-FP solutions. The time courses of *Q* and *W* were corrected according to the time-dependent spectral changes in the control (nonradioactive) samples; the values of *Q^rel^* and *W^rel^* were calculated and plotted vs. the time of exposure to HTO. The experimental errors for *Q^rel^* and *W^rel^* did not exceed 8%.

## 3. Results and Discussion

### 3.1. Effect of Tritium on the Bioluminescence System of Coupled Enzymatic Reactions: Bacterial Luciferase–NAD(P)H:FMN-Oxidoreductase

**Figure 3:**
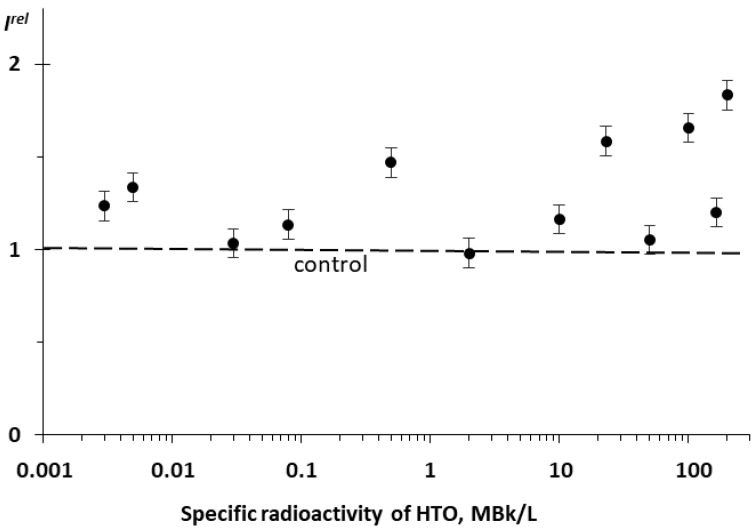
Relative bioluminescence intensity, *I^rel^*, of the coupled system of enzymatic reactions: bacterial luciferase–NAD(P):FMN-oxidoreductase vs. specific radioactivity of HTO at 20 min exposure.

We studied the effect of tritiated water, HTO, on the bioluminescence system of the coupled enzymatic reactions: bacterial luciferase–NAD(P)H:FMN-oxidoreductase. This system is supposed to model the intracellular light-emitting processes in luminous marine bacteria [54,55]. The bioluminescence intensity at different radioactivities of the media is presented in Figure 3. One can see that no monotonic dependency of *I^rel^* was observed on the HTO activity concentration; the effect was of a stochastic character with the predominant activation of the bioluminescence. This response of the enzyme system mirrors that of luminous bacteria at the initial stage of their lifetime, as shown in Figure 1B for the 20 h exposure.

In the current experiment, we did not observe any inhibitory effects of HTO. However, previous results [24] demonstrated not only the activation but also the inhibition of the bioluminescence of the coupled enzymatic system by tritium.

Therefore, luminous bacteria at initial stages of exposure (Figure 1B [16]) and the bacterial system of the coupled enzyme reactions (Figure 3) demonstrated similar bioluminescence responses to tritium. The conclusion can be made that the coupled enzymatic reactions contribute to the adaptive response of the bacteria. The responses of the individual enzymatic reactions to the similar exposure are considered below.

### 3.2. Effect of Tritium on the Enzymatic Activity of Bacterial Luciferase

**Figure 4:**
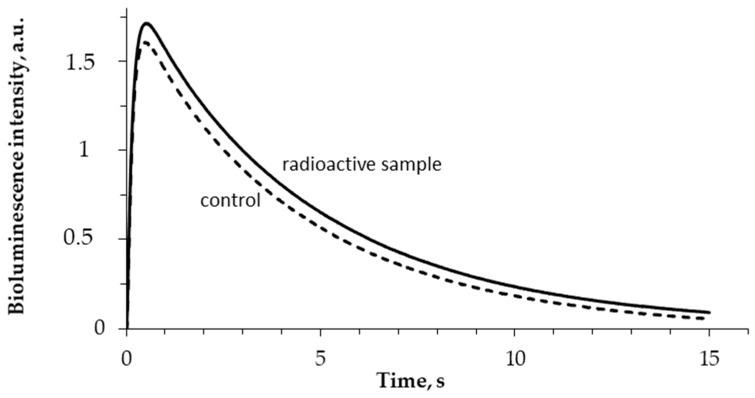
Bioluminescence kinetics in the reaction catalyzed by bacterial luciferase. 5 min incubation; 5 MBq/L specific radioactivity of HTO.

The enzymatic activity of bacterial luciferase (R1) was studied after enzyme incubation in HTO solutions under different specific radioactivities. The bioluminescence kinetic curves in the control and radioactive samples were recorded; examples of the curves are presented in Figure 4. One can see that tritium slightly increases the bioluminescence intensity in the course of the luciferase reaction.

**Figure 5:**
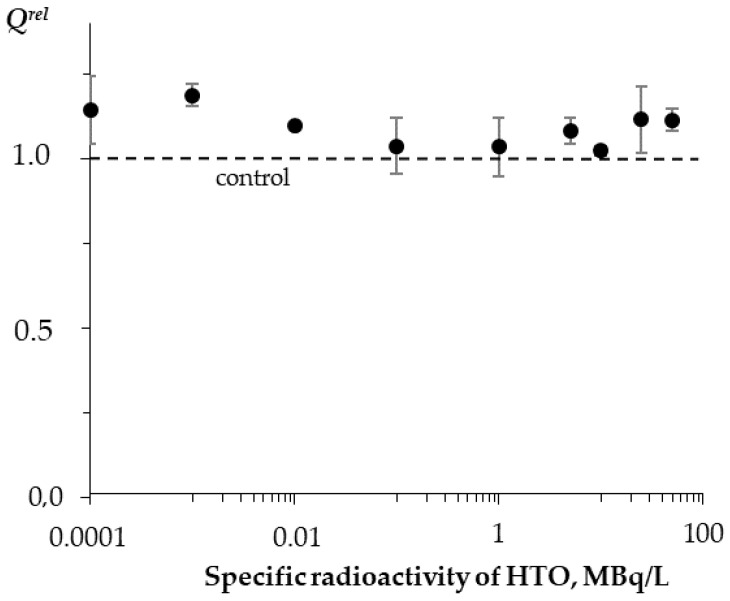
Relative activity of bacterial luciferase, *Q^rel^*, vs. specific radioactivity of HTO at 5 min exposure.

Figure 5 shows that no monotonic dependence of *Q^rel^* on the solution’s radioactivity was observed. The average *Q^rel^* values slightly exceeded 1 in all of the used HTO radioactivity range; the deviations from the control did not exceed 20%. Hence, HTO did not significantly increase the bioluminescence quantum yields in the reaction of bacterial luciferase when compared to the nonradioactive (control) samples.

The slight increase in the bioluminescence yield can probably be associated with the optimization of the structure of luciferase through the ionization of the medium in the course of the low-energy radioactive tritium decay.

Additionally, the time of incubation of bacterial luciferase with HTO (2 MBq/L) varied from 1 to 87 h. It was found that such an incubation did not noticeably change the parameters of the luciferase reaction (data not shown).

Thus, we demonstrated the independence of the luciferase enzymatic activity from the HTO radioactivity and time of the radiation exposure. The exposures did not increase the luciferase activity by more than 20%. Nevertheless, this increase contributed to the bioluminescence activation of the coupled system of the enzymatic reactions bacterial luciferase–NAD(P)H:FMN-oxidoreductase presented before in Section 3.1.

### 3.3. Effect of Tritium on the Enzymatic Activity of NAD(P)H:FMN-Oxidoreductase

**Figure 6:**
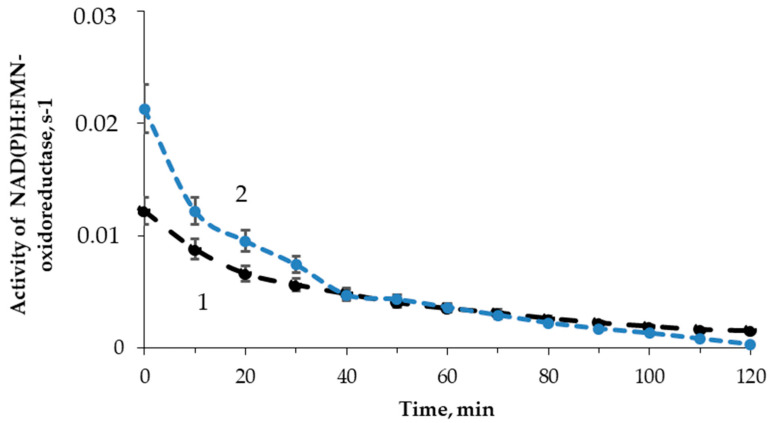
Activity of NAD(P)H:FMN-oxidoreductase vs. time of exposure to HTO: 1—control (nonradioactive) sample; 2—radioactive sample, 10 MBq/L.

The effect of tritium on the enzymatic reaction catalyzed by NAD(P)H:FMN-oxidoreductase was studied; the enzymatic activity was measured with intervals of 10 min for 2 h. Figure 6 shows examples of the enzymatic activity time courses in the control and radioactive samples. The maximal dose accumulated by the sample of 200 MBq/L radioactivity was ~2 mGy, which was much lower than the tentative limit of a low-dose interval (about 100 mGy, according to [56]).

**Figure 7:**
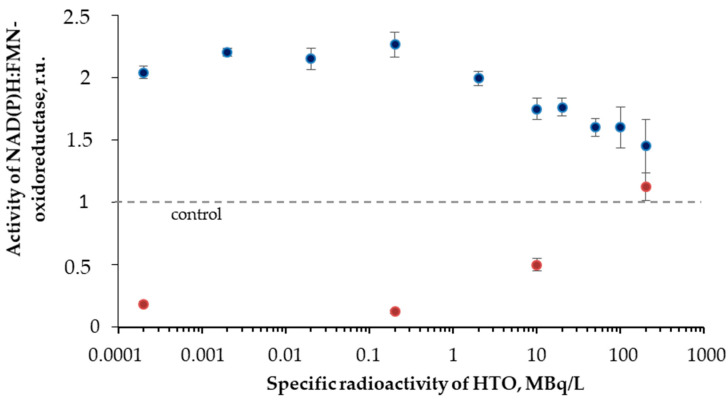
Activity of NAD(P)H:FMN-oxidoreductase vs. HTO specific radioactivity. Time of exposure was 1 min (blue) and 110 min (red).

Figure 6 shows that the initial stage of the reactions (<40 min) reveals higher enzymatic activities in the radioactive sample as compared to the control. The final stage of the reaction showed an inhibition of the enzyme activity. The solutions with other radioactivities demonstrated similar results. The illustration of the HTO effects at the initial and final stages of the reaction is presented in Figure 7 for different HTO radioactivities.

The figure shows an increase (up to 230%) and decrease of the oxidoreductase activity at 1- and 110-min exposures to HTO, respectively. It is evident that oxidoreductase is more sensitive to tritium than bacterial luciferase (Section 3.2). Since oxidoreductase supplies the bioluminescence reaction of bacterial luciferase with the substrate, *FMN∙H***^−^** (R2 and R1, respectively), the coupling of these enzymatic reactions can be responsible for the effects of tritium on the bioluminescence of the system of the coupled enzyme reactions (Figure 4) and luminous bacteria (Figure 1B). Since the bacterial cells produce this enzyme endogenously [57], it provides the long-term effects of the radionuclide on the bioluminescence intensity.

### 3.4. Effects of Tritium on Coelinteramide-Containing Fluorescent Protein

Tritium bioeffects were studied using the simplest enzymatic process, i.e., the photobiochemical reaction of Coelinteramide-containing Fluorescent Protein (CLM-FP). Previously, the low-dose exposure of CLM-FP to tritium was studied by Petrova et al. [49]. No activation was found, but only an inhibition effect of tritium: the 18-day chronic exposure to HTO resulted in an increase in the violet fluorescence component contribution. This effect revealed a decay in the efficiency of the photobiochemical reaction in CLM-FP, likely due to protein destruction in the presence of tritium. The tritium activity concentration was 200 MBq/L; the maximal accumulated dose (0.28 Gy) was close to the tentative border of the low-dose range. The work by Petrova et al. used the CLM-FP preparation with a ratio of the “violet” to the “green” fluorescence intensity (415 and 500 nm maxima, respectively) equal to 0.3.

**Figure 8:**
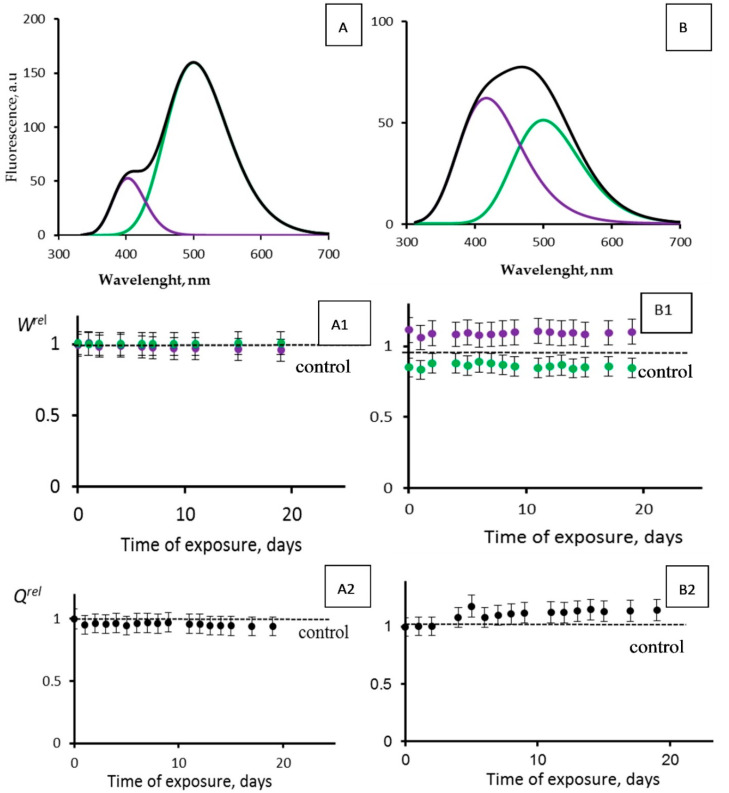
Photoluminescence spectra (*λ_ext_* = 350 nm) of CLM-FP with (**A**,**B**) different contributions of violet and green components. Black line corresponds to overall spectra. (**A1**,**B1**) Violet (violet dots) and green (green dots) fluorescence contributions, *W^rel^*, and (**A2**,**B2**) fluorescence quantum yields, *Q^rel^*, vs. time of exposure to tritiated water, 200 MBq/L.

We looked for an activation of the photobiochemical reaction of CLM-FP by tritium. To find this effect, we varied the initial fluorescence characteristics of the CLM-FP preparation by its exposure to a high temperature (40 °C) for 60 h according to [45]. The ratios of the violet to the green components’ fluorescence intensities for the chosen CLM-FP samples differed from those reported in [49]; they were ca. 0.2 and 0.8 (Figure 8A,B, respectively), detecting a different efficiency of the proton transfer in the photobiochemical reaction of CLM-FP. We assumed that the partly thermo-damaged CLM-FP samples could be more sensitive to tritium, providing the conditions for the tritium activation effect.

Figure 8 presents the photoluminescence spectra of CLM-FPs (samples A and B), as well as the results of their chronic exposure to tritiated water, HTO (Figure 8(A1,A2,B1,B2)). The maximal accumulated dose was 14 mGy. Sample A did not demonstrate any evident changes from the control: the violet and green fluorescence contributions *W^rel^* (Figure 8(A1)) and overall fluorescence quantum yields *Q^rel^* (Figure 8(A2)) did not show any deviations from those for the control samples during the observation course (19 days). Sample B demonstrated slight deviations of *W^rel^* from those for the control samples: Figure 8(B1) shows insignificant (10–15%) changes of the violet and green contributions; a slight increase in the violet component contribution and decrease in the green one indicated a decay of the efficiency of the proton transfer, likely due to an additional protein destruction by HTO. Hence, we did not find any rise of efficiency for the photobiochemical reaction of the proton transfer. These results are similar to those obtained earlier [49] with other CLM-FP samples that were not subjected to a preliminary destructive treatment. The results confirmed the conclusion [49] that the simplest bioassay system based on CLM-FP does not demonstrate an activation of photobiochemical processes under the conditions of a low-dose exposure to HTO.

An insignificant (10–15%) rise of the overall fluorescence quantum yields *Q^rel^* is evident from Figure 8(B2). This effect might be explained from a photophysics point of view: in the course of the tritium radioactive decay, the rigidity of the fluorophore structure in the CLM-FP might rise due to the ionization of the medium. The ionic strength increase might result in the electrostatic stabilization of the fluorophore and, hence, in a decrease of the nonradiative efficiency in the fluorescence emitter.

## 4. Conclusions

The hormesis phenomenon is usually attributed to organisms. Nevertheless, a study of molecular processes that provide an activation of physiological functions in organisms is crucial to understanding this phenomenon. The present study considers one of the simplest cellular organisms, a marine bacterium, and its enzymatic reactions. Additionally, the simplest enzymatic reaction, photochemical proton transfer in coelenteramide-containing fluorescent protein, was also under consideration. We compared the activation effects of the beta-emitting radionuclide tritium on the cellular organism and on the enzymatic reactions. It has been found that tritium increases the activity of NAD(P)H:FMN-oxidoreductase at the initial stage of its reaction. Through the coupling (via reduced FMN) with the bioluminescence reaction of bacterial luciferase, this reaction probably contributes to the escalation of the bioluminescence of the coupled system NAD(P)H:FMN-oxidoreductase—bacterial luciferase, resulting in the further activation of the bacterial light emission. Additionally, it has recently been shown [58] that the bacterium environment influences its response: natural bioactive compounds, humic substances, were found to mitigate the adaptive response of the bacteria to the low-dose ionizing radiation of tritium. The variation of the bacterial response in complex water solutions in the presence of redox-active compounds (such as phenolic substances including microbial secondary metabolites) [59] is a question of special interest.

An intracellular increase of the oxidoreductase activity caused by tritium might be a more complicated mechanism, as tritium can activate bacterial luminescence without penetrating into the cells [33], probably through the ionization of water and activation of membrane processes. This could be a subject of additional investigations. A comparison of the role of reactive oxygen species in cellular [32] and enzymatic processes in HTO should be provided in further studies as well.

The isolated reaction of bacterial luciferase showed a negligible activation by tritium, and no increase in the rates of the photobiochemical proton transfer in the fluorescent protein was observed. Hence, it is evident that a lower level of organization of living matter, without conjugation with other processes, hardly facilitates an activation of biological processes and related adaptive responses in multicellular organisms.

## Abbreviations

CLM	Coelenteramide
FP	Fluorescent Protein
ROS	Reactive Oxygen Species
FMN	Flavin mononucleotide
NADH	Nicotinamide adenine dinucleotide
HTO	Tritiated water
